# ECM Remodeling in Direct Inguinal Hernia: The Role of Aging, Oxidative Stress, and Antioxidants Defenses

**DOI:** 10.3390/clinpract15120219

**Published:** 2025-11-24

**Authors:** John Dawi, Yura Misakyan, Edgar Gonzalez, Kevin Kafaja, Scarlet Affa, Kevin Tumanyan, Kyla Qumsieh, Vishwanath Venketaraman

**Affiliations:** 1 College of Osteopathic Medicine of the Pacific, Western University of Health Sciences, Pomona, CA 91766, USA; john.dawi@westernu.edu (J.D.); edgar.gonzalez@westernu.edu (E.G.); kevin.kafaja@westernu.edu (K.K.); kyla.qumsieh@westernu.edu (K.Q.); 2 Department of Biochemistry and Chemistry, The College, UCLA, 405 Hilgard Avenue, Los Angeles, CA 90095, USA; yura.misakyan@westernu.edu (Y.M.); scarleta@ucla.edu (S.A.); 3 College of Podiatric Medicine, Western University of Health Sciences, Pomona, CA 91766, USA; kevin.tumanyan@westernu.edu

**Keywords:** inguinal hernia, extracellular matrix, collagen, oxidative stress, antioxidant defense

## Abstract

Inguinal hernia represents a multifactorial condition driven by extracellular matrix (ECM) dysregulation, collagen imbalance, and oxidative stress. Across studies, a consistent reduction in the collagen I:III ratio, coupled with altered expression of matrix metalloproteinases (MMPs) and tissue inhibitors of metalloproteinases (TIMPs), underpins weakened fascia and hernia susceptibility. Aging further impairs ECM remodeling through fibroblast senescence, cross-linking deficits, and elastic fiber attrition, while oxidative stress and inflammation amplify tissue degradation and impair repair mechanisms. Evidence from clinical and experimental studies underscores the interplay between surgical technique, mesh choice, redox balance, and recurrence risk. Understanding the combined impact of aging and oxidative stress provides a mechanistic framework for targeted therapeutic and surgical strategies aimed at preventing hernia development and recurrence.

## 1. Introduction

Inguinal hernia is increasingly recognized as a disorder of extracellular matrix (ECM) remodeling rather than a purely mechanical defect. A consistent observation across histological and molecular studies is an altered balance between collagen types I and III. Normally, collagen type I provides tensile strength, while type III confers elasticity. A reduction in the collagen I:III ratio predisposes fascia to structural weakness. Early investigations into hernia sac tissue demonstrated altered synthesis of collagen I and III, alongside changes in fibronectin and matrix metalloproteinase (MMP) expression, indicating active remodeling at the hernia site [1]. Further molecular studies confirmed that primary inguinal hernia patients exhibit significantly decreased procollagen I:III mRNA ratios, accompanied by upregulated MMP-1 and MMP-13 activity [2]. These findings suggest that hernia development involves both diminished structural collagen and accelerated matrix degradation. In recurrent inguinal hernia, the imbalance is even more pronounced, with tissue samples showing persistently low collagen I:III ratios and elevated collagenolytic enzyme activity, implicating a chronic ECM disorder that undermines repair durability [3].

The relevance of collagen imbalance is not confined to the hernia sac tissue. Peeters et al. (2014) demonstrated that skin biopsies from hernia patients mirror the collagen I:III ratio observed in fascia, with a strong correlation (*r* = 0.81), suggesting that systemic connective tissue alterations can be detected in accessible peripheral tissues [4]. Additional work on tissue inhibitors of metalloproteinases (TIMPs) revealed decreased TIMP-2 expression in fascia transversalis of hernia patients, further shifting the balance toward excessive collagen breakdown [5]. Collectively, these data are supported by broader reviews, which conclude that inguinal hernia arises from a pathological ECM phenotype marked by abnormal collagen turnover, MMP/TIMP imbalance, and age-related connective tissue degeneration [6].

Superimposed on these intrinsic matrix vulnerabilities is the role of oxidative stress and inflammation, which can further destabilize ECM integrity. Polat et al. (2003) compared open versus laparoscopic hernia repair and found significantly higher oxidative stress markers, such as malondialdehyde and protein carbonyls, after open repair, suggesting that surgical trauma and technique influence redox balance [7]. Randomized trials of mesh implantation have also highlighted oxidative and immune responses: Donati et al. (2016) demonstrated that heavy polypropylene meshes provoked stronger oxidative stress, including lower glutathione levels and higher lipid hydroperoxides, compared with lightweight meshes [8].

More recent work has refined this perioperative redox picture. Białecki et al. (2020) reported that single-incision laparoscopic totally extraperitoneal (TEP-SI) repair elicited significantly lower oxidative stress than multi-port TEP repair, indicating that less invasive access reduces systemic oxidative load [9]. Similarly, Yang et al. (2024) found that laparoscopic TEP repairs decreased inflammatory mediators and oxidative stress markers compared with open surgery, potentially contributing to faster recovery and improved tissue healing [10].

**Figure 1:**
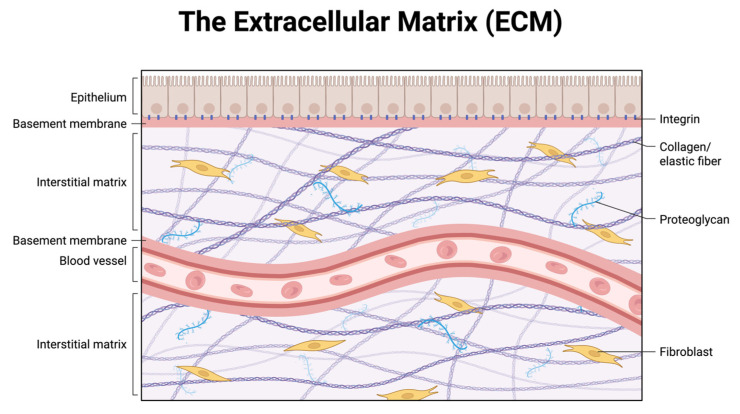
Organization of the extracellular matrix (ECM) in abdominal wall fascia. Schematic showing epithelium, basement membrane, and interstitial matrix with key components (collagen/elastin fibers, proteoglycans) and cells (fibroblasts) linked via integrins.

Taken together, these findings support a two-hit model of inguinal hernia pathogenesis: intrinsic connective tissue weakness driven by collagen I:III imbalance and dysregulated ECM remodeling, compounded by extrinsic oxidative and inflammatory stresses that further degrade tissue integrity. This dual framework not only explains the higher recurrence rates seen in older and systemically vulnerable patients but also underscores the importance of tailoring surgical technique and implant selection to minimize redox stress and preserve ECM stability. To orient the reader, the abdominal wall extracellular matrix is illustrated schematically, highlighting its major compartments (basement membrane and interstitial matrix), key macromolecules (collagen/elastin fibers, proteoglycans), and fibroblast–integrin linkages (Figure 1).

Although this review integrates biochemical, genetic, and clinical data into a unified redox-driven model of inguinal hernia, it is important to recognize that much of the current evidence remains correlational or mechanistic rather than causal. Most studies linking oxidative stress, MMP activation, and collagen imbalance derive from in vitro fibroblast assays, animal models, or small observational cohorts analyzing hernia sac tissue. Large-scale, prospective trials directly testing redox-modulating interventions—such as antioxidant therapy, metabolic optimization, or mesh bio-functionalization—are still lacking. Moreover, while molecular findings demonstrate biological plausibility, they cannot yet define quantitative risk thresholds for clinical translation. Future studies should therefore employ longitudinal and interventional designs to clarify whether correcting oxidative imbalance or enhancing ECM stability can prevent hernia formation or reduce recurrence rates in high-risk populations. Until such data become available, the proposed model should be viewed as a mechanistically grounded framework for hypothesis generation rather than definitive clinical proof.

## 2. Collagen and Extracellular Matrix Biology

Type I and type III collagens form the tensile backbone of the abdominal wall. Type I, with thick, highly cross-linked fibrils, provides stiffness and load-bearing strength, whereas type III, with thinner fibrils, confers compliance and is enriched during repair and in more distensible tissues. The balance between them—the type I:type III ratio—sets tissue mechanics and failure thresholds [11,12].

Collagen molecules assemble as triple helices that laterally associate into quarter-staggered fibrils; fibril diameter and spacing are regulated during and after secretion, not merely by synthetic rate [13,14]. Early fibrillogenesis is scaffolded by fibronectin microfibrils and integrin signaling (especially *α5β1*), which coordinate cell traction with matrix assembly and align nascent collagen into load-bearing cables [15,16,17]. After deposition, covalent cross-linking by the lysyl-oxidase (LOX/LOXL) family stabilizes fibrils and determines stiffness and creep resistance. Inhibition or insufficiency of LOX yields mechanically weak matrices with abnormally shaped, polydisperse fibrils despite normal collagen content—evidence that cross-linking is a quality, not quantity, control on collagen [18,19]. Matrix remodeling is continuous: collagenases (MMP-1/-13) and gelatinases (MMP-2/-9) degrade fibrillar and denatured collagen, while TIMPs (tissue inhibitors of metalloproteinases) restrict that proteolysis; dysregulated MMP/TIMP activity shifts net collagen toward loss and disorganization [20,21].

Small leucine-rich proteoglycans (SLRPs) such as decorin and lumican bind fibril surfaces and precisely tune fibril diameter, spacing, and lateral fusion. Gain- or loss-of-function of these “molecular calipers” alters collagen architecture without changing total collagen, again emphasizing organization over mass [22,23,24].

Hernia-relevant human data map onto these core ECM principles. Biochemical analyses of groin hernia tissue show altered collagen composition consistent with a reduced type I:type III ratio and abnormal remodeling, supporting a systemic or at least field-defect process in the abdominal wall [25]. In the transversalis fascia—a key load-bearing layer—fibroblasts from patients with direct inguinal hernia constitutively overexpress active MMP-2, indicating a pro-degradative phenotype that persists ex vivo and is not solely reactive to in vivo cues [26]. Similar up-regulation of active MMP-2 with relative reduction in TIMP-2 is detectable in abdominal skin fibroblasts from hernia patients, consistent with a broader connective-tissue susceptibility beyond the hernia margin [27]. Conversely, direct hernia fascia shows decreased TIMP-2 immunostaining compared to controls and indirect hernia, a shift that would favor sustained MMP-2 activation and collagen loss [5].

Compositionally, fascia from patients with indirect hernia can also show collagen perturbations—both type I and III reduced, with a proportionally greater fall in type I—again pointing to an unfavorable I:III balance even when total collagen is not increased [28]. Practical corollary: the skin collagen I:III ratio correlates with abdominal wall fascia, suggesting minimally invasive sampling could surrogate the deeper field [4]. Broader clinical observations support a connective-tissue basis: recurrent hernia has been framed as a disorder of the collagen matrix, with recurrent cases demonstrating more pronounced derangements in collagen content and remodeling markers [3].

Finally, cross-link biology appears relevant in hernia. LOXL1—an elastin/collagen cross-linking enzyme—shows reduced expression in direct hernia fascia together with increased elastase, implying impaired elastic-fiber integrity and suboptimal cross-link maturation within the load path of the groin. Such a deficit would lower yield strength even at unchanged collagen mass, harmonizing with the MMP/TIMP evidence toward a net mechanically inferior ECM [29]. Collectively, the mechanistic picture is consistent: integrin/fibronectin-guided deposition, SLRP-tuned fibril architecture, LOX-mediated cross-linking, and tightly regulated MMP/TIMP turnover together maintain a collagen network with a high type I:type III ratio and robust cross-link quality. In inguinal hernia—especially the direct subtype—human fascia and skin show a reproducible tilt toward degradation (↑MMP-2, ↓TIMP-2), lower cross-link competency (↓LOXL1), and an unfavorable shift in the type I:type III balance. These are the precise ingredients of a matrix that stretches, thins, and ultimately fails under physiologic load [11,12,13,14,15,16,17,18,19,20,21,22,23,24,25,26,27,28,29].

## 3. Aging and Extracellular Matrix Remodeling

Aging reshapes fascia by eroding the cell–matrix dialog that normally preserves tensile collagen, tightening control of proteolysis, and maintaining elastic recoil; the end result is a matrix whose composition (lower type I:III), organization (fragmented fibrils, inferior cross-linking), and mechanics (loss of recoil, higher stress concentrations) all tilt toward failure.

### 3.1. Fibroblast Mechanics → Signaling → Collagen Output

Chronological aging reduces type I collagen synthesis by human dermal fibroblasts even when those cells are placed onto permissive matrices, indicating a cell-intrinsic loss of anabolic drive and defective mechano-responsiveness (reduced stimulation by normal physical cues) [30]. Diminished cell spreading and contractile force—termed “fibroblast collapse”—shrinks cytoskeletal tension, which is a primary upstream driver of collagen transcription in healthy tissue [31]. In aged human skin, fragmentation of surrounding collagen weakens integrin anchorage, elevates intracellular oxidative stress, and upregulates MMP-1, closing a feed-forward loop in which fragmentation begets more fragmentation [32]. In parallel, CTGF/CCN2—a key TGF-*β*-responsive cofactor for collagen production—falls in chronologically aged skin, offering a concrete transcriptional link between aging and collagen loss [33]. Causality is testable: forcing catalytically active MMP-1 expression in dermal fibroblasts is sufficient to fragment collagen and reproduce the structural and functional hallmarks of aged ECM (impaired attachment, altered morphology, reduced matrix deposition) [34].

Mechanics are not bystanders; reduced cell area/force directly up-regulates MMP-1 via c-Jun/AP-1, accelerating fibril fragmentation in vitro [35]. In vivo confirmation shows smaller fibroblasts in aged human skin with concordant elevation of multiple MMPs, anchoring a geometry-to-protease axis in natural aging [36]. The same mechanical downshift reduces TGF-*β* type II receptor (T*β*RII) abundance, blunting canonical pro-collagen signaling and locking tissues into a low-synthesis state, even when ligands are present [37].

### 3.2. Senescence Programs Sustain a Degradative Milieu

Aging increases the burden of senescent fibroblasts that cease proliferation but secrete the senescence-associated secretory phenotype (SASP)—a cocktail of IL-6, IL-8, TNF-*α*, growth factors, and MMPs that amplifies local inflammation, stimulates proteolysis, and perturbs repair; while tumor-suppressive in intent, chronic SASP erodes collagen and elastin and promotes non-resolving remodeling in aging stroma [38].

### 3.3. Cross-Link Quality and Glycation Damage

Beyond quantity, collagen quality declines with age. Non-enzymatic glycation accumulates advanced glycation end-products (AGEs) on long-lived collagens, stiffening fibrils, reducing elasticity, and impairing normal proteostasis; AGEs also engage RAGE signaling, feeding oxidative stress and MMP induction—molecular currents that further degrade ECM [39]. Reconstructed-skin models show that glycation alone reproduces key features of aged matrix—fragmented, disorganized collagen, weaker fibroblast adhesion, and diminished tissue integration—demonstrating a direct, cell-external mechanism for aging-like degradation [40].

### 3.4. Elastic Fiber Attrition and Cross-Link Enzyme Drift

Elastic fibers—an elastin core wrapped by fibrillin microfibrils—age by fragmentation, calcification, and loss of recoil, shifting load to collagen and amplifying strain on already compromised fibrils [41]. Reviews across tissues confirm that aging and disease accelerate elastin degradation and dysfunctional remodeling, undermining recoil and increasing mechanical fatigue in connective tissues [42]. In hernia-prone fascia specifically, LOXL1 (a cross-linking enzyme essential for both elastin and collagen maturation) is downregulated, while elastase activity is elevated, a signature of impaired elastic-fiber repair and inferior cross-link maturation within the transversalis fascia load path [31].

### 3.5. Human Abdominal Wall Evidence for Age-Linked Collagen Shifts

Comparative peritoneal studies show adults have lower collagen type I:III ratios than infants, mapping an age-associated compositional drift toward a more compliant, weaker matrix in hernia-relevant tissues [43]. In the transversalis fascia of patients with indirect inguinal hernia, total collagen I and III are both reduced, but type I decreases disproportionately, yielding an unfavorable I:III ratio that predicts lower tensile competence [28]. At the molecular level, fibroblasts derived from primary hernia patients show reduced type I:type III procollagen mRNA ratios and heightened MMP-1/-13 expression, consistent with a constitutively degradative phenotype [2]. Similar imbalance of type I and III mRNAs is demonstrable in incisional hernia fibroblasts, underscoring that connective-tissue fragility and pro-degradative programming persist ex vivo and are not solely local surgical artifacts [44].

**Figure 2:**
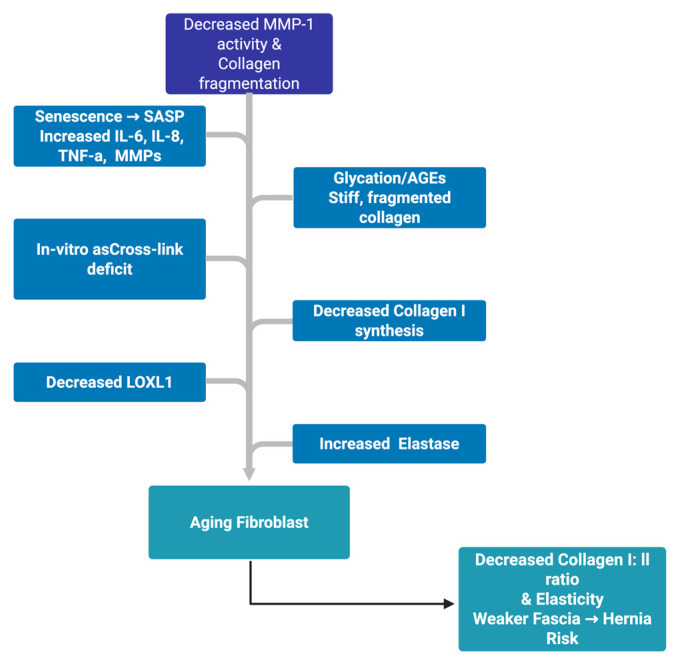
**Drivers of fibroblast aging and weakened abdominal wall fascia.** Senescence with a SASP profile (IL-6, IL-8, TNF-*α*, MMPs), non-enzymatic glycation/AGEs, impaired cross-linking (LOXL1), decreased collagen I synthesis, and increased elastase converge on the aging fibroblast. The net effect is a lower collagen type I: III ratio and reduced elasticity, resulting in weaker fascia and an increased risk of hernia.

Abdominal wall biology syntheses converge on a systems model: with age, tissues transition from a high-tension, collagen-dominant, well-cross-linked network to a milieu of low fibroblast force, higher MMP burden, inferior cross-linking (glycation or LOXL1 drift), and elastin loss, yielding thinner, disorganized collagen with a lower type I: III ratio (Figure 2).

### 3.6. Integrative View and Clinical Implications

Abdominal wall biology syntheses converge on a systems model: with age, tissues transition from a high-tension, collagen-dominant, well-cross-linked network to a milieu of low fibroblast force, higher MMP burden, inferior cross-linking (glycation or LOXL1 drift), and elastin loss, yielding thinner, disorganized collagen with a lower type I:III ratio [45,46]. Large-scope review work places advanced age, aberrant collagen metabolism, and ECM genetic/biologic susceptibility among the most consistent risk factors for hernia development and recurrence, integrating cellular mechanics, matrix chemistry, and hereditary predisposition into one etiologic arc [47]. Matrix changes in hernia fascia-reduced collagen I: III, higher MMP activity, and lower TIMP (Table 1).

## 4. Oxidative Stress and Inflammation

Oxidative stress and inflammation form a central biochemical landscape in inguinal hernia pathophysiology and repair. The processes involve reactive oxygen species (ROS) damaging proteins and lipids, lipid peroxidation products such as malondialdehyde (MDA) and TBARS accumulating, and depletion of endogenous antioxidant defenses like superoxide dismutase (SOD), catalase, and glutathione (GSH). In parallel, inflammatory mediators such as interleukin-6 (IL-6), tumor necrosis factor-*α* (TNF-*α*), and calprotectin rise sharply, activating leukocytes and fibroblasts to secrete more proteases. Evidence across human clinical trials confirms that oxidative and inflammatory perturbations are present both preoperatively in hernia patients and amplified by surgery and mesh implantation.

### 4.1. Oxidative Stress Differences Between Open and Laparoscopic Repair

Open hernia repair induces a stronger oxidative burden than minimally invasive techniques. In a controlled clinical comparison, both laparoscopic and open repair caused rises in serum MDA (a stable marker of lipid peroxidation), but the rise was significantly higher in open repairs. Simultaneously, erythrocyte SOD activity fell more sharply in open cases, reflecting exhaustion of the enzymatic antioxidant system [10]. These biochemical changes paralleled patient outcomes: patients in the laparoscopic group experienced less postoperative pain, quicker mobilization, and shorter hospital stays, showing that lower oxidative stress correlates with faster recovery.

TBARS (thiobarbituric acid reactive substances) provided additional resolution. In a trial comparing tension-free mesh repair with the older Andrew’s suture technique, postoperative TBARS were significantly lower in the tension-free group [7]. Reduced tissue tension and ischemia during repair likely account for this finding: less ischemia means less reperfusion-driven ROS.

Longitudinal studies show that IL-6 rises within 6 h after hernia repair and peaks at 24 h, while oxidative markers peak earlier, suggesting that ROS generation is a trigger for subsequent cytokine cascades [48,49,50]. Laparoscopy consistently results in lower IL-6 and TNF-*α* peaks, and lower MDA and TBARS levels, than open repair [10,48,50]. This points to an integrated loop: smaller incisions and less manipulation → reduced ROS generation → blunted cytokine surge → faster resolution of inflammation.

In the most refined comparison, single-incision laparoscopic totally extraperitoneal (SILTEP) repair was tested against conventional multi-port TEP. Patients in the SILTEP group had significantly lower total oxidative status (TOS) and oxidative stress index (OSI), and better preservation of total antioxidant capacity (TAC) [50]. The clinical translation was reduced pain scores and quicker return to activity, directly linking oxidative biochemistry to outcomes.

### 4.2. Mesh Implantation and Chronic Oxidative-Inflammation Balance

Polypropylene mesh provokes a foreign body reaction in which macrophages and neutrophils release ROS and cytokines. A randomized trial compared lightweight vs. heavyweight polypropylene meshes: the heavyweight mesh group had significantly higher lipid hydroperoxides, reduced GSH levels, and elevated IL-6 and TNF-*α* at early postoperative time points [9]. Lightweight meshes preserved antioxidant status better and induced lower cytokine release, consistent with a smaller oxidative footprint.

Mesh pore size and architecture also matter. In another prospective analysis, smaller-pore, denser polypropylene meshes caused a stronger leukocytic infiltrate, higher early postoperative leukocyte counts, and greater cytokine secretion compared with larger-pore meshes [8]. The proposed mechanism is that tighter mesh structures entrap more macrophages, which generate ROS through NADPH oxidase, amplifying local oxidative injury and potentially promoting collagen degradation at the implant–fascia interface.

These mesh-related oxidative processes may persist long after the acute phase, as polypropylene degradation products are themselves oxidatively active and capable of perpetuating macrophage activation. Thus, mesh selection is not merely mechanical but biochemical, influencing the redox environment of fascia during healing.

### 4.3. Systemic Inflammatory Mediators and Anesthesia Modulation

The systemic inflammatory profile of hernia surgery includes both classical cytokines and novel innate immune markers. IL-6 and CRP rise after both open and laparoscopic repair, but the magnitude is greater after open procedures [48,49]. IL-6 is strongly associated with postoperative fatigue, while CRP correlates with systemic recovery time.

Anesthetic choice further modulates oxidative stress. A prospective study of patients undergoing Lichtenstein repair under local, spinal, or general anesthesia showed that lipid peroxidation (MDA) was highest under general anesthesia and lowest under local anesthesia. Concurrently, SOD and catalase activities were best preserved under local anesthesia, indicating stronger antioxidant defense [51]. A larger randomized trial confirmed that IL-6 and CRP were lowest under local anesthesia, intermediate under spinal, and highest under general anesthesia [52]. The explanation likely lies in differences in systemic neuroendocrine and immune activation across anesthetic modalities.

Novel markers add further granularity. Calprotectin, a neutrophil-derived mediator, rises within 3–6 h after hernia surgery and peaks at 12 h, declining thereafter [53]. This rapid rise makes it a sensitive early marker of innate immune activation, potentially superior to CRP, which peaks later. Importantly, calprotectin kinetics align with neutrophil-driven ROS bursts, reinforcing the link between innate immune activity and oxidative stress.

### 4.4. Preoperative Oxidative Stress in Hernia-Prone Patients

Even outside the surgical setting, oxidative stress appears to be part of the hernia phenotype. In patients with inguinal hernia and joint hypermobility syndrome, plasma TOS was significantly elevated, TAS was reduced, and the oxidative stress index (OSI) was higher compared with controls. At the same time, prolidase activity—an enzyme essential for recycling proline from degraded collagen—was significantly increased [54]. Elevated prolidase suggests accelerated collagen turnover under oxidative pressure, consistent with a chronic imbalance between collagen degradation and synthesis. This constitutional oxidative profile likely contributes to fascia fragility and may predispose patients to hernia formation independent of surgical stress.

### 4.5. Broader Surgical Evidence: Oxidative Stress as a General Principle

The oxidative burden of hernia repair parallels other abdominal procedures. In patients undergoing cholecystectomy or lower abdominal surgery (including hernia repairs), plasma MDA, 8-isoprostane, and lipid hydroperoxides all rose postoperatively, with greater increases after open compared with laparoscopic surgery [55]. Laparoscopic patients preserved antioxidant capacity better, consistent with reduced tissue ischemia–reperfusion and less systemic trauma. This confirms that oxidative stress is a generalizable surgical phenomenon, and hernia repair fits squarely within this framework.

### 4.6. Synthesis and Clinical Implications

A systematic review consolidates these findings: across dozens of studies, hernia surgery induces systemic oxidative and inflammatory stress measurable by MDA, TBARS, lipid hydroperoxides, TOS/TAS, GSH depletion, SOD activity loss, IL-6, TNF-*α*, CRP, and calprotectin [56]. The amplitude of response is dictated by:

- Surgical technique: open > laparoscopic > single-incision laparoscopic
- Mesh type: heavy/low-pore > lightweight/high-pore
- Anesthesia: general > spinal > local

These oxidative and inflammatory dynamics matter clinically. Fascia in elderly patients already has reduced type I collagen, poor cross-linking, and fibroblast senescence (as shown in Section 3). Superimposed oxidative stress further degrades collagen, activates MMPs, and depletes antioxidant defenses, creating a biochemical environment where repairs are more likely to fail and recurrences more likely to occur. Thus, oxidative and inflammatory stress are not just acute byproducts of surgery but determinants of long-term hernia biology and recurrence risk.

## 5. Antioxidant System and Redox Control of ECM Remodeling

Oxidative stress (excess reactive oxygen/nitrogen species, ROS/RNS) and the capacity of endogenous antioxidant systems (glutathione, glutathione peroxidase, glutathione reductase, superoxide dismutase, catalase) form a biochemical “gain knob” on ECM remodeling: they tune collagen synthesis vs. degradation, the activation state of matrix metalloproteinases (MMPs), and the behavior of fibroblasts that maintain fascia. In inguinal hernia, clinical studies show measurable perturbations of redox markers around surgery and with specific patient phenotypes, while mechanistic work demonstrates how glutathione-centric signaling governs collagen balance and MMP activation [57].

### 5.1. Clinical Redox Signatures Around Inguinal Hernia and Repair

Minimally invasive access lowers the oxidative burden: single-incision TEP (TEP-SI) generates a smaller systemic oxidative response than multi-port TEP, reflected by lower lipid peroxidation (e.g., TBARS) and a more favorable total antioxidant status (TAS), indicating reduced perioperative ROS production and better preservation of the antioxidant buffer [57]. Mesh characteristics modulate this biology: heavier polypropylene meshes drive a stronger inflammatory–oxidative response (higher IL-6, TNF-*α*, lipid hydroperoxides; lower glutathione), consistent with a higher redox load on the healing fascia and a shift toward degradative remodeling [9]. Outside the intraoperative setting, patient phenotype also matters: children with inguinal hernia who have joint hypermobility display higher oxidative stress and elevated prolidase activity, a pro-turnover signal consistent with enhanced collagen breakdown pressure [8]. Laparoscopic repair can attenuate the postoperative inflammatory/oxidative surge compared with open techniques, which correlates with faster recovery kinetics and a biochemical milieu that is friendlier to constructive ECM remodeling [10,52]. Together, these data imply that surgical approach and implant choice are not merely technical decisions; they are levers on the peri-fascial redox environment that will either stabilize or erode collagen architecture in the early phases of healing [10,57,58].

### 5.2. Redox Control Points for Collagen Balance and MMP Activity

Glutathione (GSH), the dominant low-molecular-weight antioxidant in cells, directly regulates TGF-*β*–driven collagen synthesis in fibroblasts: adequate GSH permits robust TGF-*β*–stimulated matrix production, while GSH depletion blunts this anabolic response [7]. GSH also governs the “other side” of collagen turnover: in fibroblasts that have been pushed into a TGF-*β*–dominated state (pro-synthesis, anti-degradation), replenishing glutathione can restore collagen degradation capacity, indicating that redox tone sets the switch position between build and clear [58]. Beyond glutathione’s permissive role on synthesis and degradation programs, oxidant signaling tunes both pro-collagen expression and MMP activity: in cardiac fibroblasts (a canonical connective-tissue model), increased ROS enhances MMP expression/activity while altering collagen synthesis, mapping a generalizable redox-to-remodeling axis that is highly relevant to abdominal wall fascia [59].

At the post-translational level, oxidants modify MMPs and their regulators via protein S-glutathionylation and other redox-sensitive cysteine switches. Peroxynitrite (a potent RNS) drives S-glutathiolation-dependent activation of MMPs, which tilts the ECM balance toward proteolysis even without de novo transcriptional upregulation [60]. More broadly, redox-driven S-glutathionylation of signaling and structural proteins is a reversible code that integrates oxidative cues with matrix turnover, thereby linking the oxidative burst of surgery and inflammation to real-time collagen degradation potential [61]. These mechanistic “redox rheostats” explain why small shifts in perioperative ROS/RNS and GSH buffers can have large effects on fascia stability [58,59,60,61].

### 5.3. The Glutathione Enzyme Network and Antioxidant “Throughput”

The glutathione system is not just GSH concentration; it is a cycle with dedicated enzymes that set antioxidant throughput. GSH is regenerated from its oxidized form (GSSG) by glutathione reductase (GR) using NADPH; a sluggish GR step slows GSH recycling, lengthening the time the cell spends in an oxidized state and narrowing the safety margin against ROS bursts [62]. Glutathione peroxidase-1 (GPx1) reduces hydrogen peroxide and lipid hydroperoxides at the expense of GSH, thereby preventing secondary propagation of lipid peroxidation and membrane/protein damage; insufficient GPx1 activity amplifies lipid-derived radicals and feeds back into MMP activation pathways [63,64]. At the signaling layer, protein S-glutathionylation acts as a reversible post-translational modification that protects sensitive cysteines from irreversible oxidation while modulating enzyme activities and transcriptional circuits relevant to ECM homeostasis—this is a key logic gate connecting redox status to gene programs and protease control [65,66]. A fascia with robust GSH/GR/GPx capacity can tolerate transient ROS/RNS spikes without flipping into a sustained proteolytic, collagen-fragmenting state; a fascia with a thin GSH buffer or low GPx1 becomes “noisy,” favoring MMP activation and poor collagen preservation [62,63,64,65,66].

### 5.4. Mechanistic Synthesis for the Hernia Fascia

Integrating the clinical and mechanistic strands, a consistent picture emerges. The perioperative oxidative surge transiently pushes the GSH/GSSG ratio downward, increasing S-glutathionylation events and enabling redox-activation of latent MMPs, especially in tissues already primed by inflammation or heavier meshes [9,60,61,66]. Lower antioxidant reserve (TAS, GSH, GPx1 activity) correlates with higher lipid peroxidation (TBARS/LOOH), which not only damages the matrix but also amplifies protease networks through redox signaling [61,63,64]. Phenotypic susceptibility—as seen in joint hypermobility—co-travels with elevated oxidative markers and prolidase activity, suggesting a collagen-turnover axis that can be tipped further by surgical and implant choices [8]. At the fibroblast level, GSH availability sets the TGF-*β* rheostat for collagen synthesis and degradation: depleted GSH blunts collagen production and, paradoxically, can also lock tissues into a “can’t-clear-the-damage” mode unless GSH is restored [7,58]. In sum, redox control is not an epiphenomenon—it is a first-order driver of whether fascia heals toward restored I:III strength or spirals into fragmentation and laxity [7,61,62,63,64,65,66].

### 5.5. Practical Implications for Trials and Practice

These data support a perioperative redox strategy alongside surgical optimization: (i) prefer access/techniques that minimize oxidative surge (TEP-SI where appropriate) [66]; (ii) select meshes with lower inflammatory–oxidative profiles, especially in high-risk patients (older age, redox-sensitive comorbidities) [9]; (iii) monitor a biomarker panel—TAS, GSH/GSSG, GPx1 activity, TBARS/LOOH, and MMP activity—to identify patients who remain in a pro-oxidant/proteolytic state post-repair [10,58,59,60,65,66]; (iv) consider targeted redox interventions in trials (nutritional or pharmacologic augmentation of GSH/GPx1/GR capacity) with prespecified endpoints of MMP activation and collagen integrity to test whether shifting the redox set-point improves ECM outcomes [7,58,65,66]. By embedding redox biology into technique and biomarker-guided care, we turn a passive risk factor into an active therapeutic axis for inguinal hernia biology [10,57,58,59,60,65,66].

## 6. Synergy Between Aging and Oxidative Stress (“Double-Hit Pathogensis”)

**Figure 3:**
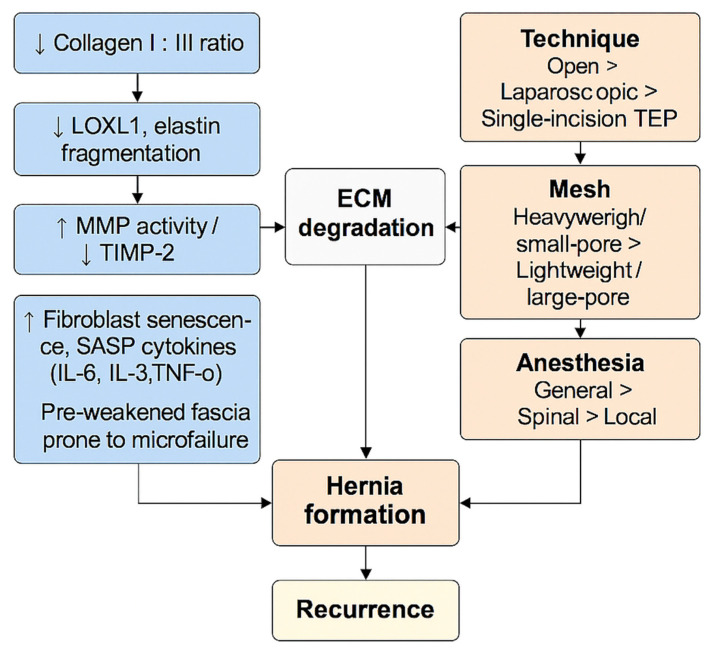
**The double hit hypothesis summary flow chart.** This figure shows that hernias result from a mix of biological weakness and surgical factors. On the biological side, a lower collagen I to III ratio, reduced LOXL1, increased MMP activity, and fibroblast aging all break down the extracellular matrix. This leaves the fascia weakened and more likely to fail. On the surgical side, choices such as the repair technique, the mesh used, and the type of anesthesia can add mechanical stress or inflammation. Both pathways lead to extracellular matrix degradation, hernia formation, and eventually recurrence.

Aging and oxidative stress do not act in parallel; they interlock as shown in Figure 3. Aging establishes a collagen architecture that is easier to damage, which is characterized by a lower type I:III balance and fibroblasts biased away from constructive remodeling; while oxidative stress supplies the biochemical push (ROS/RNS, mitochondrial injury, redox-sensitive protease activation) that fragments fibrils, sustains matrix metalloproteinase (MMP) activity, and suppresses repair. In hernia-prone fascia, this coupling yields a self-reinforcing loop: older tissue fails sooner under everyday loads, and oxidative signaling prevents recovery, converting small microfailures into clinical defects.

### 6.1. Aging “Primes” the ECM for Failure

Multiple human studies demonstrate that hernia tissue displays a systemic shift toward collagen type III and away from type I, weakening tensile properties before any acute oxidative insult occurs. Skin and transversalis fascia from inguinal hernia patients show increased collagen III in skin and decreased collagen I in fascia, lowering the I:III ratio in both layers—evidence of a field defect rather than a purely local abnormality [67]. At the gene-expression level, fibroblasts derived from primary hernia patients exhibit reduced type I:type III procollagen mRNA ratios together with MMP signals that favor matrix turnover [68]. Cell-culture work further shows the intrinsic nature of this phenotype: hernia-derived fibroblasts retain a markedly lowered *α*1(I):*α*1(III) collagen ratio ex vivo, indicating a programmatic bias toward type III synthesis independent of immediate tissue context [2]. Biochemical analyses of adult groin hernias corroborate abnormal collagen content/solubility in fascia, consistent with altered assembly and maturation that would reduce load-bearing strength [67]. Together, these data define the first “hit”: aging-aligned ECM remodeling that produces a more compliant, fracture-prone collagen network [67].

### 6.2. Oxidative Stress Accelerates Breakdown and Blocks Repair

Aging simultaneously compromises mitochondrial homeostasis and redox defenses, amplifying the impact of ROS/RNS on matrix integrity. Mitochondrial dysfunction with age increases electron-transport leakage and ROS generation, which damages mitochondrial DNA and proteins; the resulting ROS–mtDNA injury cycle perpetuates oxidative signaling that spills into extracellular remodeling [25]. In aged human cells, the response to ROS is impaired at multiple nodes—antioxidant buffering, proteostasis, and damage clearance—producing greater protein aggregation and a slower return to redox baseline after stress [69]. In connective tissue, that biology maps to higher MMP activity, greater collagen fragmentation, and slower reconstitution of a stable I:III balance once injury initiates [25,69].

### 6.3. Convergence in Hernia Tissue: Skin Mirrors Fascia; Recurrence Magnifies the Phenotype

Because the “aging” phenotype is systemic, skin collagen I:III ratios closely track those in abdominal wall fascia (*r* ≈ 0.81), allowing a peripheral window into the load-bearing layer’s biology and underscoring that the primed state is body-wide rather than focal [70]. When oxidative/inflammatory hits accumulate (surgery, chronic strain, comorbidities), tissues with this baseline collagen vulnerability progress to recurrent failure: explants from patients with recurring inguinal/incisional hernias display markedly reduced collagen I:III ratios compared with controls, consistent with sustained turnover and poor repair [4]. These observations operationalize the “double-hit” model: aging sets the fragile baseline; oxidative stress pushes the matrix over the edge and keeps it there [4,69,70].

### 6.4. Mechanistic Map of the Double-Hit Loop

1. Baseline weakness (aging): lower collagen I:III, abnormal maturation/solubility, and fibroblasts biased toward type III and matrix turnover [67].
2. Oxidative push: mitochondrial ROS amplifies MMP signaling and inhibits efficient collagen re-synthesis; aged cells clear oxidative damage poorly, prolonging the degradative window [25,69].
3. Systems coupling: skin–fascia coupling means systemic aging signals are registered in the abdominal wall; recurrent cases reflect repeated traversal of this loop, with progressively lower I:III and stiffer, more fragmented matrices [4,70].

Net effect: in older fascia, everyday mechanical loads generate microtears that cannot be neutralized because oxidative signaling keeps MMPs “on” and fibroblasts “off,” preventing restoration of a high-I:III, strongly cross-linked network. Clinically, this explains why age increases initial hernia risk and recurrence, and why interventions that damp oxidative stress or bolster antioxidant capacity plausibly shift the trajectory back toward constructive repair [4,67].

## 7. Intracellular Signaling Pathways Involved in ECM Remodeling Under Oxidative Stress

### 7.1. Nrf2/Keap1 Pathway and ECM Remodeling Under Oxidative Stress

The Nrf2 (nuclear factor erythroid 2–related factor 2) pathway is the cell’s master defense system against oxidative stress. Under homeostatic conditions, Nrf2 is sequestered in the cytoplasm by Keap1, which targets it for ubiquitin-mediated degradation. When oxidative stress increases, reactive cysteine residues on Keap1 become oxidized, causing conformational changes that release Nrf2. Freed Nrf2 translocates into the nucleus, where it binds to antioxidant response elements (AREs) in the promoters of detoxifying and cytoprotective genes, including glutathione peroxidase (GPx), superoxide dismutase (SOD), heme oxygenase-1 (HO-1), and NAD(P)H quinone oxidoreductase 1 (NQO1) [71].

In fibroblasts, activation of the Nrf2 pathway preserves ECM homeostasis by reducing intracellular ROS, inhibiting NF-κB–driven MMP expression, and maintaining a pro-synthetic phenotype. Studies in skin and pulmonary fibroblasts show that Nrf2 activation decreases MMP-1 and MMP-9 levels while sustaining type I collagen synthesis, counteracting oxidative damage–induced fragmentation of the ECM. Conversely, Nrf2 depletion amplifies fibroblast senescence, impairs wound healing, and drives the shift toward a catabolic, collagen-degrading phenotype seen in aging fascia [71].

Mitochondrial integrity is another Nrf2-dependent checkpoint: Nrf2 controls mitochondrial biogenesis via transcriptional upregulation of PGC-1*α* and TFAM, and it suppresses mitochondrial ROS leakage, which are both crucial for maintaining fibroblast energy metabolism required for collagen secretion. These mechanisms highlight how Keap1-Nrf2 imbalance contributes to the “oxidative exhaustion” of connective tissues, where reduced antioxidant gene expression accelerates ECM degradation, cross-link disruption, and susceptibility to hernia formation [71].

Nrf2’s role extends beyond detoxification; it interfaces with the TGF-*β*/Smad and NF-κB networks, buffering against their pro-fibrotic and pro-inflammatory extremes. This crosstalk positions Nrf2 as the molecular “rheostat” that determines whether oxidative stress leads to adaptive remodeling or chronic degradation [71].

### 7.2. NF-κB Pathway: Oxidative Stress, MMP Activation, and Collagen Breakdown

The NF-κB pathway is one of the principal redox-sensitive signaling cascades linking oxidative stress to extracellular matrix (ECM) degradation. In quiescent fibroblasts, NF-κB dimers (usually p65/p50) are held inactive in the cytoplasm by the inhibitor IκB. Reactive oxygen species (ROS) generated by mitochondrial leakage, UV exposure, or chronic inflammation activate the IκB kinase complex, causing IκB phosphorylation and proteasomal degradation. The liberated NF-κB then translocates into the nucleus, where it binds promoter regions of genes encoding matrix metalloproteinases (MMP-1, MMP-3, MMP-9) and inflammatory cytokines such as IL-6 and TNF-*α*, which together accelerate matrix turnover.

Human fibroblast and skin studies demonstrate that chronic oxidative stress keeps NF-κB constitutively active, maintaining high levels of MMP expression and inhibiting the synthesis of structural collagens. Fisher and colleagues showed that NF-κB activation in aged and photo-oxidatively stressed skin is accompanied by robust MMP-1 expression and collagen fibril fragmentation, confirming that redox imbalance directly drives ECM degradation [72]. Reviews on human skin aging further identify NF-κB as part of a sustained oxidative-inflammatory loop—ROS activate NF-κB, which up-regulates MMPs, whose activity generates additional oxidative by-products that reinforce NF-κB signaling [73].

Mechanical stress can amplify this process. In fibroblasts embedded within three-dimensional collagen matrices, matrix tension alone activates NF-κB and increases MMP-1 expression, linking the physical strain of connective tissues to biochemical signaling that weakens their architecture [74]. Together, these findings establish NF-κB as a redox-mechanical integrator: transient activation promotes repair, but chronic stimulation—by oxidative stress, aging, or mechanical overload—locks fascia into a self-perpetuating state of inflammation and proteolysis, undermining its tensile strength and predisposing to hernia formation.

### 7.3. TGF-β/Smad and FoxO Signaling Under Oxidative Stress: Redox-Sensitive Remodeling of the Extracellular Matrix

The transforming growth factor-*β* (TGF-*β*) signaling system is the core regulatory axis of fibroblast activity and connective-tissue remodeling. Under physiological conditions, latent TGF-*β* complexes are released from the extracellular matrix (ECM) through proteolysis or integrin-mediated mechanical strain, events enhanced by reactive oxygen species (ROS) [1]. Activated TGF-*β* binds the type II receptor (T*β*RII), recruiting and phosphorylating TβRI/ALK5, which in turn phosphorylates Smad2/3. The Smad2/3–Smad4 complex then translocates to the nucleus, inducing transcription of COL1A1, COL1A2, and TIMP-2, while suppressing MMP-1 and MMP-9, thereby promoting collagen synthesis and organized fibrillogenesis [75].

Under oxidative stress, this balance deteriorates. ROS both activate and are generated by TGF-*β* signaling, forming a feed-forward loop in which TGF-*β* induces NOX4-derived superoxide and H_2_O_2_, and these oxidants further amplify Smad and MAPK pathways [76,77]. In normal fibroblasts, transient ROS bursts fine-tune Smad activity, but chronic oxidative exposure sustains the pathway abnormally, pushing cells toward myofibroblast differentiation and maladaptive ECM remodeling. ROS oxidize phosphatases that normally deactivate TGF-*β* kinases, keeping the pathway constitutively active. In parallel, non-Smad arms such as p38-MAPK and JNK phosphorylate Smad linker regions, altering gene-target specificity and promoting matrix degradation instead of synthesis [75,76,77].

Direct experimental evidence in human skin fibroblasts demonstrates that sustained oxidative exposure reduces T*β*RII and Smad3 protein levels, thereby impairing canonical Smad signaling and suppressing type I collagen expression [78]. These redox-dependent receptor losses shift fibroblasts toward a catabolic phenotype, where ECM turnover is high but the mechanical integrity of collagen is poor. The redox-blunted TGF-*β* response described in vitro closely parallels histologic findings in aging fascia, where fibroblasts exhibit low p-Smad3, high oxidative load, and impaired fibrillogenesis—features strongly associated with hernia susceptibility [77,78].

Cellular glutathione (GSH) acts as the major rheostat of this system. GSH depletion augments TGF-*β*-driven CTGF and collagen expression through ROS-dependent mechanisms, while pharmacologic restoration of GSH rebalances Smad signaling and limits fibrogenic gene expression [79]. When antioxidant defenses are intact, fibroblasts favor constructive remodeling; when exhausted, the TGF-*β*/ROS axis degenerates into persistent remodeling without structural repair—a key biochemical hallmark of degenerative fascia [79].

The Forkhead box O (FoxO) transcription factors—particularly FOXO1 and FOXO3—operate as intracellular sentinels counteracting this oxidative drift. Normally, Akt phosphorylation sequesters FoxO in the cytoplasm via 14-3-3 proteins, suppressing its transcriptional activity. Under oxidative stress, JNK and AMPK phosphorylation and SIRT1-mediated deacetylation reactivate FoxO by promoting its nuclear localization [80,81]. Once reactivated, FoxO drives the expression of SOD2, catalase, peroxiredoxin, and GSH-synthesizing enzymes, thereby restoring redox balance. It also induces autophagy-related genes such as LC3 and BNIP3, preserving proteostasis and mitochondrial quality control [80,81,82].

Through these antioxidant and proteostatic effects, FoxO indirectly preserves ECM integrity. By reducing ROS accumulation, FoxO attenuates NF-κB activation and the downstream surge of MMPs that otherwise degrade fibrillar collagen. When FoxO activity declines—owing to sustained Akt signaling or reduced SIRT1 deacetylation with aging—fibroblasts experience increased oxidative load, inefficient collagen secretion, and exaggerated matrix degradation [81,82]. FoxO and TGF-*β* thus form a redox-responsive dyad: FoxO suppresses TGF-*β*-induced ROS production, while persistent TGF-*β* signaling represses FoxO expression through Smad interference, reinforcing a vicious cycle of oxidative stress and ECM fragility [75,76,77,78,79,80,81,82].

The combined dysregulation of TGF-*β*/Smad and FoxO pathways, therefore, underlies a unified pathophysiological model of hernia formation. Chronic oxidative stress tilts TGF-*β* from a regenerative to a destructive mode while eroding FoxO-mediated antioxidant control. The fascia remains metabolically active but structurally ineffective—producing collagen that is chemically cross-linked yet biomechanically fragile. Over time, this redox-driven connective-tissue exhaustion culminates in the progressive weakening of the transversalis fascia, the anatomic substrate of inguinal hernia.

### 7.4. P38-MAPK Pathway in Oxidative Stress–Driven ECM Remodeling

The p38 mitogen-activated protein kinase (p38-MAPK) cascade serves as a crucial redox-sensitive node in connective-tissue biology, integrating oxidative stress signals into transcriptional and post-transcriptional control of extracellular matrix (ECM) turnover. Activation begins when upstream kinases MKK3 and MKK6 phosphorylate p38*α*/*β* at threonine 180 and tyrosine 182. Reactive oxygen species (ROS) enhance this process by directly oxidizing redox-sensitive cysteine residues on MAPK kinases and by sustaining their activity through the inactivation of phosphatases such as MKP-1 [83]. Once activated, p38 phosphorylates a wide array of substrates, including ATF-2, CREB, and Elk-1, which collectively promote transcription of inflammatory and matrix-degrading genes.

In fibroblasts, oxidative stress and transforming growth factor-*β* (TGF-*β*) signaling converge upon the p38 pathway to regulate collagen and protease expression [84]. TGF-*β* can induce type I collagen synthesis through p38-dependent mechanisms, but chronic activation shifts the equilibrium toward ECM degradation rather than repair. Under persistent oxidative stress, p38 prolongs the stability of MMP-1 and MMP-3 mRNAs through post-transcriptional mechanisms mediated by HuR and TTP, extending their half-lives and thereby sustaining proteolytic activity within the fascia [85]. This sustained MMP expression results in the disorganization of collagen fibrils, diminished cross-linking, and the progressive mechanical weakening characteristic of degenerative fascial tissue.

Recent studies in fibroblast and skin models confirm that ROS-driven p38 activation underlies tissue stiffening, collagen fragmentation, and aberrant remodeling typical of aging connective tissue. For instance, oxidative exposure markedly increases p38 phosphorylation and downstream MMP-1 and MMP-3 expression in human dermal fibroblasts, leading to ECM disorganization and wrinkle formation—molecular phenomena that closely parallel fascial weakening in hernia pathogenesis [86]. Conversely, pharmacologic inhibition of p38 or antioxidant interventions such as N-acetylcysteine restore collagen organization and normalize fibroblast contractility [87].

Taken together, p38-MAPK constitutes the biochemical fulcrum through which oxidative stress converts adaptive repair into chronic degeneration. By coupling ROS production to Smad linker phosphorylation, mRNA stabilization, and fibroblast senescence, this pathway produces a state of metabolic hyperactivity with mechanical failure—a defining biological substrate of hernia-prone fascia [83,84,85,86,87].

### 7.5. MicroRNA Control of ECM Remodeling Under Oxidative Stress: miR-21 (Pro-Fibrotic) and miR-29 (Anti-Fibrotic)

Signal direction and upstream control. Oxidative stress and TGF-*β* signaling converge on microRNA programs that rewire fibroblast behavior. miR-21 is consistently induced by TGF-*β*1 in primary human fibroblasts and in fibrotic tissues; its up-regulation amplifies TGF-*β* signaling by repressing negative regulators (e.g., Smad7, PTEN) and by promoting myofibroblast differentiation with heightened collagen synthesis and contractility [88,89,90]. Antagonizing miR-21 in vivo reduces experimental fibrosis and dampens fibroblast pro-fibrotic activity, establishing miR-21 as a redox/TGF-*β*–responsive driver of connective-tissue remodeling [88]. In parallel, the miR-29 family (miR-29a/b/c) is broadly suppressed by TGF-*β*1 across fibrotic models; this suppression removes brakes on a large cohort of ECM genes (e.g., COL1A1, COL1A2, COL3A1, ELN, FBN1), thereby shifting fibroblasts toward a matrix-deposition state even as oxidative cues promote protease expression [91,92,93,94].

Direct ECM targets and matrix balance. In fibroblasts, miR-21 increases type I collagen output and collagen-gel contraction while cooperating with TGF-*β* to sustain *α*-SMA–positive myofibroblast features; it also facilitates inflammatory crosstalk that feeds MMP expression (via AP-1/NF-κB), tipping turnover toward high-flux but low-quality matrix remodeling [88,89,90]. Conversely, miR-29 directly targets multiple collagen and elastin transcripts; gain-of-function reduces fibrillar collagen and elastin expression, while inhibition of miR-29 raises ELN and collagen levels—demonstrating a post-transcriptional throttle on ECM accumulation and cross-link scaffolding [93,94]. Large-scale profiling confirms that miR-29 is a master negative regulator of fibrosis-associated genes, with its downregulation correlating with disease severity across organs [92].

Integration with redox/MAPK/Smad circuitry. Oxidative stress sustains TGF-*β* and p38-MAPK activity, which together induce miR-21 and suppress miR-29, locking fibroblasts into a loop of amplified collagen gene transcription (via Smads), enhanced contractility, and increased susceptibility to protease-mediated fragmentation—because the same redox milieu drives MMP-1/3/9 through AP-1 and NF-κB. Mechanistically, this produces a paradox typical of aging fascia: more matrix is synthesized, but it is disorganized, fragment-prone, and mechanically inferior. Restoring miR-29 (or relieving its TGF-*β*–mediated suppression) re-balances the transcriptome toward lower collagen/ELN output and can blunt fibrotic progression; blocking miR-21 reduces myofibroblast activation and collagen hyperproduction while easing TGF-*β* feed-forward pressure [88,89,90]. In the hernia context, this miRNA axis explains how chronic oxidative stress can simultaneously increase collagen gene drive and worsen collagen architecture, yielding a fascia that is metabolically busy but structurally weak.

### 7.6. Circulating Biomarkers of Collagen Turnover and Oxidative Stress

The interplay between extracellular matrix (ECM) remodeling and systemic redox imbalance can be quantitatively assessed through circulating biomarkers that reflect collagen synthesis, degradation, and antioxidant capacity. Among these, the N-terminal propeptides of type I and III procollagen (PINP and PIIINP) represent established indicators of collagen turnover. PINP originates from type I collagen synthesis, whereas PIIINP reflects type III collagen formation—an isoform enriched in mechanically stressed and healing fascia. Elevated serum PIIINP has been linked with excessive fibroblast activity and matrix remodeling in fibrotic and cardiovascular disorders, making it a candidate marker of ongoing fascial degeneration [95].

Matrix metalloproteinases (MMP-2 and MMP-9) and their endogenous inhibitors (TIMP-1, TIMP-2) serve as dynamic indicators of ECM catabolism and repair balance. The MMP/TIMP ratio provides functional insight into the tissue’s proteolytic tone—values skewed toward MMP dominance indicate increased collagen degradation, while excessive TIMP activity suggests fibrotic rigidity. Circulating TIMP-1 has been validated as a sensitive systemic biomarker for pathological remodeling, often correlating with serum PIIINP and hyaluronic acid levels in fibrosis cohorts [96,97]. In hernia-prone fascia, a parallel imbalance likely reflects chronic oxidative activation of fibroblasts and MMP overexpression.

Oxidative stress can be further monitored by the glutathione redox pair (GSH/GSSG) and total antioxidant status (TAS). These parameters capture systemic oxidative burden and cellular antioxidant reserve. Reduced GSH/GSSG ratios correspond with increased ROS-driven signaling in fibroblasts, reinforcing the catabolic shift in the ECM. Combining these biochemical metrics—PINP/PIIINP for synthesis, MMP/TIMP for degradation, and GSH/GSSG for redox status—could provide an integrative, minimally invasive biomarker panel to stratify patients by connective-tissue vulnerability and to monitor the impact of antioxidant or surgical interventions over time [95,96,97].

## 8. Sex Based and Hormonal Modulation of ECM Remodeling

Sex hormones exert profound regulatory effects on extracellular matrix (ECM) homeostasis, influencing fibroblast behavior, collagen turnover, and redox signaling. Androgens have been shown to alter the expression of matrix proteins, leading to delayed wound healing and reduced collagen deposition, largely through modulation of fibroblast phenotype and MMP activity [98]. This may contribute to the higher prevalence of inguinal hernia observed in men, as androgenic signaling tends to favor ECM degradation over repair. In contrast, estrogen demonstrates a protective effect on connective-tissue integrity by enhancing tissue inhibitor of metalloproteinase (TIMP) activity and suppressing MMP expression, thereby preserving the collagen I/III ratio and supporting organized remodeling [99]. Experimental studies further indicate that estrogen-mediated pathways restore the MMP–TIMP balance, improve collagen cross-linking, and mitigate oxidative stress–driven ECM injury, effects that diminish with menopause and aging [99,100]. Moreover, cellular-level studies reveal intrinsic sex-based differences in fibroblast mechanotransduction and ECM remodeling, independent of hormonal milieu, suggesting that chromosomal and epigenetic factors may also shape connective-tissue resilience [100]. Complementary reviews on organ fibrosis show that males generally exhibit greater oxidative and fibrotic responses than females under comparable injury stimuli, underscoring a systemic redox–hormonal interplay that extends to fascial tissue remodeling [101]. Together, these findings support the concept that both sex hormones and genetic sex influence ECM stability, collagen organization, and oxidative signaling—factors that collectively modulate hernia susceptibility and healing dynamics. However, direct investigations into sex-specific ECM behavior in human hernia models remain scarce, representing a key area for future translational research.

## 9. Clinical Implications and Future Directions

### 9.1. Why Elderly Patients Recur More Often

The burden of inguinal hernia recurrence is disproportionately observed in elderly populations, but the relationship between age and recurrence is multifaceted. Registry-based analyses involving thousands of patients suggest that patient-related risk factors such as hernia type, sex, smoking, and prior recurrence are more consistent predictors of recurrence than age alone [102]. This means that while aging does not always independently predict recurrence, the older population often clusters with other risk-enhancing factors. Clinical series add nuance: in patients undergoing the Shouldice repair, older individuals experienced recurrence at a shorter postoperative interval than younger patients, pointing toward age as a modifier of recurrence timing rather than a categorical risk factor [103].

High-volume registry data confirm the impact of surgical expertise and center experience, where centers specializing in hernia surgery demonstrate lower recurrence rates across all ages [104]. Conversely, multicenter reviews highlight that the interplay of hernia morphology (direct vs. indirect), patient exposures such as smoking, and technical variability together explain much of the heterogeneity in recurrence outcomes [105]. In practice, elderly fascia is biologically weaker due to prior ECM remodeling, oxidative stress, and diminished regenerative capacity, making these patients more vulnerable to early recurrence, especially when other modifiable risk factors coexist.

### 9.2. Toward Risk Stratification: Candidate Biomarkers

Collagen I:III ratio. The most consistent tissue-level biomarker is the reduced type I:III collagen ratio, observed not only in transversalis fascia but also in skin samples from hernia patients. Since type I collagen provides tensile strength and type III collagen contributes to elasticity, an imbalance tilting toward collagen III creates a mechanically fragile fascia [106]. Importantly, skin biopsy data suggest that systemic connective tissue changes mirror local fascial remodeling, raising the possibility of minimally invasive testing [106]. Molecular studies link genetic predispositions in collagen and MMP genes to hernia formation, providing further support that collagen dysregulation underpins recurrence susceptibility [4].

Circulating peptides. Blood-based collagen turnover markers offer another promising window into ECM biology. The PINP/PIIINP ratio, reflecting the balance of mature versus immature collagen synthesis, is consistently lower in recurrent hernia patients. Proposed cut-offs for this ratio demonstrate good diagnostic accuracy for identifying recurrence-prone individuals, making it a candidate for preoperative triage or postoperative monitoring [2].

MMP/TIMP axis. Enzymatic regulators of ECM turnover are also altered in hernia patients. Serum levels of MMP-2 and TIMP-2 are elevated in both primary and recurrent cases, but especially in recurrent direct hernias, suggesting a systemic predisposition toward unbalanced collagen degradation [107]. Surgical trauma itself modulates these enzymes, as demonstrated by perioperative studies documenting transient spikes in circulating MMPs and TIMPs after repair [108]. This indicates that enzyme activity is not only a biomarker of baseline ECM vulnerability but also a dynamic indicator of healing quality.

Oxidative stress biomarkers. Hernia surgery provides a unique model to study oxidative stress in real time. Patients undergoing open Lichtenstein repair exhibit significantly higher levels of oxidative stress markers—malondialdehyde (MDA), TBARS, and protein carbonyls—along with lower antioxidant reserves than those undergoing laparoscopic repairs [109]. Laparoscopic TEP and single-incision TEP repairs are consistently associated with smaller oxidative surges and better preservation of antioxidant defenses [7]. These findings position oxidative stress panels (MDA, TBARS, carbonyls, thiol groups, TAS) as valuable markers not just of tissue injury but of surgical approach quality.

Glutathione. The reduced tripeptide glutathione (GSH) is the keystone of cellular antioxidant defense, buffering ROS and maintaining redox balance. While direct hernia-specific data are limited, reviews across chronic illnesses highlight that low GSH status strongly correlates with impaired recovery and heightened oxidative stress [9]. This suggests that preoperative GSH quantification could help identify patients lacking sufficient antioxidant reserves to mount effective ECM repair.

### 9.3. Therapeutic Strategies to Test

**Surgical technique as a redox lever**. Since oxidative stress responses differ significantly between approaches, laparoscopic repair can be seen not only as a less invasive option but also as a redox-sparing therapy. Studies comparing Lichtenstein to laparoscopic techniques consistently show lower oxidative biomarker surges in minimally invasive procedures [7,9]. For elderly patients with already compromised fascia, technique selection could directly influence ECM healing capacity.

**Mesh optimization**. Mesh choice also strongly influences the tissue environment. Implantation of lightweight polypropylene mesh induces less oxidative and inflammatory stress compared to heavyweight alternatives, with corresponding reductions in systemic and local markers of oxidative injury [10,110]. Long-term studies on explanted meshes demonstrate that polypropylene undergoes oxidative stiffening over time, which reduces compliance and may contribute to pain or recurrence. Hydrogel or antioxidant-coated meshes, already tested in preclinical and clinical studies, show promise in attenuating the foreign body response and improving long-term outcomes [10].

**MMP-targeted therapies**. Preclinical models support pharmacological modulation of ECM turnover. Doxycycline, a tetracycline-class antibiotic, reduces MMP expression, shifts the collagen balance toward type I, and improves tensile strength in hernia repair models [81]. While not yet trialed clinically, this suggests that short-course, phenotype-guided MMP inhibition could be a strategy for patients with biomarker-confirmed collagen or MMP imbalance.

**Elastin and LOX pathway support**. Beyond collagen, elastin integrity is impaired in hernia patients. Fascial biopsies demonstrate reduced expression of lysyl oxidase-like 1 (LOXL-1) and tropoelastin, combined with elevated elastase activity [8]. Since LOX enzymes cross-link elastin and collagen fibers, their dysfunction compromises both elastic recoil and tensile strength. This highlights the potential of nutritional (e.g., copper as a LOX cofactor) or pharmacological interventions to restore elastin function and fascial resilience.

**Targeted antioxidant therapy**. While nonspecific antioxidant supplementation has yielded mixed results in surgery, a biomarker-guided approach could optimize its use. Patients with low GSH or elevated oxidative stress indices (MDA, TBARS, TAS) may be selectively targeted for perioperative antioxidant regimens, reducing ECM damage and improving outcomes [7,105,106]. Emerging biomaterials, including antioxidant-coated meshes, extend this logic into implant design [8].

### 9.4. Surgical and Translational Implications: Integrating ECM Biology with Mesh Innovation and Patient Optimization

Advances in hernia repair increasingly recognize that long-term success depends not only on mechanical reinforcement but also on the biological compatibility between prosthetic material and host fascia. Traditional synthetic meshes, primarily composed of polypropylene, provide immediate tensile strength but can provoke a sustained inflammatory and oxidative response, leading to fibrosis, mesh contraction, and chronic pain. The interface between mesh fibers and the transversalis fascia becomes a site of persistent macrophage activation, reactive oxygen species (ROS) release, and matrix metalloproteinase (MMP) induction. These processes mirror the same redox-driven ECM degradation described in the pathogenesis of hernia formation itself, effectively perpetuating the cycle of oxidative injury within the repair site [111,112].

To counter these limitations, biologic and hybrid meshes have been developed using decellularized extracellular matrix (ECM) scaffolds derived from porcine or human dermis. These biologically active materials contain native collagen, glycosaminoglycans, and residual signaling peptides that promote constructive tissue remodeling rather than foreign-body fibrosis. Clinical and preclinical studies demonstrate that ECM-coated or ECM-based meshes reduce macrophage infiltration, suppress TNF-*α* and MMP-9 activity, and support organized collagen deposition at the mesh–tissue interface [32,111]. In experimental implantation models, polypropylene meshes coated with an ECM hydrogel exhibit a marked reduction in fibrotic encapsulation and a shift from pro-inflammatory M1 to reparative M2 macrophage phenotypes, indicating that bioactive surface modification can reprogram the local immune response toward regenerative remodeling [112,113].

A promising refinement of this approach involves antioxidant-coated meshes, which aim to mitigate the oxidative microenvironment generated during the foreign-body reaction. Vitamin E-coated polypropylene meshes have shown decreased lipid peroxidation, reduced neutrophil infiltration, and lower ROS generation compared with uncoated controls, without compromising tensile integrity. Such coatings directly address the reviewer’s point that oxidative stress undermines repair durability, linking biochemical homeostasis to mechanical resilience. Similarly, experimental designs incorporating nitric-oxide–releasing or polyphenol-functionalized coatings are being explored to modulate inflammation and stimulate angiogenesis, thereby promoting balanced ECM remodeling at the repair site [114].

Beyond prosthetic innovation, the biological quality of host tissue remains a decisive determinant of outcome. Conditions such as diabetes mellitus, chronic obstructive pulmonary disease, obesity, and sarcopenia impair antioxidant defense systems, heighten systemic inflammation, and alter fibroblast metabolism, leading to delayed or suboptimal mesh integration. This insight has given rise to the concept of “biological prehabilitation”; a perioperative strategy aimed at improving redox balance and tissue integrity before surgery. Optimization may include glycemic control, smoking cessation, correction of malnutrition, and supplementation with antioxidant-rich nutrients such as vitamin C, vitamin E, or N-acetylcysteine, all of which support collagen synthesis and limit ROS accumulation. Integrating these measures into surgical planning reframes hernia repair from a purely mechanical intervention into a bioengineering process that combines material science, redox physiology, and clinical optimization [111,112,113,114,115].

Recent clinical data reinforce the concept that surgical outcomes in fascial reconstruction depend on the biological quality of the tissue substrate as much as on the mechanical technique employed. Fernicola and colleagues reported a monocentric experience of 31 patients with abdominal wall endometriosis who underwent wide excision and reconstructive repair following chronic inflammatory damage [105]. Their analysis demonstrated that tissue integrity, oxidative stress burden, and the extent of ECM disorganization were decisive determinants of postoperative stability. When reconstructive planning accounted for both defect geometry and fascial biology, long-term durability markedly improved.

Notably, Fernicola et al. emphasized that tissues chronically exposed to inflammation and oxidative degradation exhibit reduced collagen cross-linking, altered fibroblast phenotype, and increased MMP activity, paralleling the same redox-mediated mechanisms described in inguinal hernia. They observed that mesh selection tailored to local tissue conditions, particularly the use of biologic or hybrid prosthetics that integrate with native fascia, yielded superior functional outcomes and fewer recurrences compared with purely synthetic reinforcement. This work elegantly illustrates the principle of an ECM-aware surgical philosophy, where understanding the molecular state of the fascia (collagen integrity, redox status, and fibroblast viability) guides not only material choice but also patient optimization before and after surgery.

By integrating these clinical observations into the broader redox framework of hernia pathophysiology, the concept of “biological readiness” emerges: successful repair requires mechanical precision, but also restoration of the tissue’s biochemical competence. The inclusion of the Fernicola et al. study thus strengthens the translational continuity between molecular pathology, material science, and clinical practice, confirming that fascial biology, not prosthetic strength alone, ultimately determines repair durability [116].

## 10. Genetic Predisposition and Connective-Tissue Vulnerability in Inguinal Hernia

Growing evidence indicates that susceptibility to inguinal hernia is partly encoded within the genome, reflecting inherited differences in collagen architecture and extracellular matrix (ECM) maintenance. A single-nucleotide polymorphism (SNP) in COL1A1—the Sp1-binding site variant (rs1800012)—has been associated with increased risk of primary inguinal hernia. This variant enhances transcriptional activity of the *α*1(I) collagen gene, altering the normal *α*1:*α*2 chain ratio and producing fibrils with abnormal stiffness and reduced tensile resilience [117]. Transcript analyses from hernia sac tissue also reveal up-regulation of COL1A1 and COL3A1 together with MMP-2, suggesting an imbalance between matrix synthesis and degradation that may be genetically programmed rather than purely mechanical [118].

Large-scale genome-wide association studies (GWAS) support a broader polygenic model. A multi-cohort analysis identified susceptibility loci near ELN, FBLN5, and LOX, genes that govern elastic-fiber assembly and collagen cross-linking, confirming that hernia risk aggregates with loci mediating ECM homeostasis [119]. LOX and its paralogs encode lysyl oxidases required for covalent cross-links that stabilize fibrillar collagen and elastin. Functional studies show that LOX deficiency or polymorphism reduces cross-link density, weakening connective tissue and promoting aneurysm formation in vascular models—a mechanistic parallel to fascial attenuation in hernia [120,121].

The biological continuum between systemic connective-tissue disorders and hernia further strengthens this genetic argument. Patients with Ehlers–Danlos syndrome or cutis laxa, which feature mutations in collagen or elastin genes, display high rates of abdominal-wall defects, underscoring that localized hernia may represent a milder phenotype of generalized ECM fragility. Thus, while mechanical strain initiates fascial deformation, inherited variants in structural or regulatory ECM genes determine the threshold at which tissue failure occurs.

## 11. Methods

This review was conducted as a narrative synthesis with structured elements of a scoping review, designed to integrate molecular, genetic, and clinical data regarding extracellular matrix (ECM) remodeling, oxidative stress, and aging in the pathogenesis of inguinal hernia. The objective was not to quantify pooled outcomes but to construct a biologically coherent model linking redox imbalance to fascial degeneration.

**Databases and Search Approach**
A comprehensive literature search was performed between January 2000 and August 2025 using PubMed, Scopus, and Web of Science. Additional references were identified from the bibliographies of retrieved articles and key reviews. Search terms were combined using Boolean operators (“AND,” “OR”) and adapted for each database. The principal search string included:
(“inguinal hernia” OR “abdominal wall hernia” OR “fascial weakness”) AND (“extracellular matrix” OR “collagen remodeling” OR “oxidative stress” OR “aging” OR “fibroblast senescence” OR “matrix metalloproteinase” OR “MMP” OR “TIMP” OR “redox” OR “Nrf2” OR “TGF-beta” OR “FoxO” OR “MAPK”).

**Inclusion and Exclusion Criteria**

**Included studies met the following criteria:**
Published in English between 2000 and 2025.
Investigated ECM composition, collagen metabolism, oxidative stress, or fibroblast biology in the context of hernia, connective-tissue disorders, or abdominal wall reconstruction.
Derived from experimental (in vitro/in vivo), observational, or clinical research providing mechanistic or translational insight.

**Excluded studies were:**
Non-peer-reviewed abstracts, conference proceedings, or anecdotal case reports without molecular or histological data.
Publications lacking relevance to connective-tissue remodeling (e.g., purely procedural or epidemiological without biological correlation).

**Data Categorization and Evidence Stratification:**
Evidence was categorized into three hierarchical domains:
Experimental (molecular and animal models examining oxidative stress, ECM signaling, or fibroblast senescence).
Observational (human histological or biochemical studies of hernia sac or fascia).
Clinical and Translational (studies on biomarkers, surgical materials, and outcome correlations).

Each source was critically appraised for methodological clarity and mechanistic relevance. Findings were synthesized qualitatively to integrate molecular pathways with clinical phenomena, aligning the structure of this review with contemporary frameworks for narrative-scoping hybrid analyses.

## 12. Conclusions

Inguinal hernia represents more than a localized structural defect—it is the clinical expression of a systemic imbalance between oxidative stress, extracellular matrix (ECM) remodeling, and cellular repair. The evidence consolidated across molecular, genetic, and translational studies suggests that hernia formation is not simply mechanical “wear and tear,” but a biochemical failure of collagen homeostasis driven by chronic redox imbalance. Within this framework, reactive oxygen species (ROS) serve as both triggers and amplifiers: they activate TGF-*β*, NF-κB, and p38-MAPK pathways, induce MMPs, and destabilize collagen cross-linking, gradually eroding the tensile integrity of fascia. At the cellular level, fibroblast senescence and diminished FoxO and Nrf2 signaling reduce antioxidant defense and alter the transcriptional balance between collagen synthesis and degradation. Genetic polymorphisms in COL1A1, COL3A1, and LOX further lower the structural threshold for failure, while down-regulation of anti-fibrotic regulators such as miR-29 and up-regulation of miR-21 reinforce a pro-oxidant, remodeling-dominant phenotype. The consequence is a fascia that remains metabolically active but mechanically weak—continuously turning over matrix proteins without achieving durable reinforcement.

Clinically, this redox-centric model reframes hernia management as a biological–mechanical continuum rather than a purely surgical event. Durable repair requires not only an optimal prosthetic scaffold but also a biologically competent substrate. Meshes coated with extracellular matrix hydrogels or antioxidant polymers have demonstrated the capacity to dampen the local inflammatory milieu, reduce ROS production, and guide regenerative collagen alignment. Simultaneously, patient-level interventions—glycemic control, nutritional optimization, smoking cessation, and antioxidant supplementation—restore systemic redox balance, improving the integration and longevity of the repair. This synthesis underscores a paradigm shift: successful hernia surgery depends as much on biological insight as on mechanical technique. Understanding inguinal hernia as a redox-driven model of connective-tissue degeneration not only unites diverse strands of evidence—from molecular signaling to mesh engineering—but also invites a new generation of therapies aimed at restoring oxidative equilibrium, modulating fibroblast behavior, and rebuilding the fascia on its biochemical as well as structural foundations.

## Figures and Tables

**Table 1:** Key extracellular matrix differences in normal vs. hernia fascia.

Features	Normal Fascia	Herniated Fascia
Collagen Balance	Collagen I > Collagen III	Decreased Collagen I Increased Collagen III
Protease Activity	MMP activity balanced	Increased MMP-1, -13, -2
Protease Inhibition	Adequate TIMP-2	Decreased TIMP-2
Outcome	Stable Fibrils, Strong ECM	Weakened ECM leading to increased hernia susceptibility

## Data Availability

No new data were created or analyzed in this study.
